# Cysteamine administration in lambs grazing on mountain pastures: Effects on the body weight, antioxidant capacity, thyroid hormones and growth hormone secretion

**DOI:** 10.1002/vms3.644

**Published:** 2021-09-29

**Authors:** Borhan Shokrollahi, Abdullah Fazli, Salim Morammazi, Nazila Saadati, Hafiz Ishfaq Ahmad, Faiz‐ul Hassan

**Affiliations:** ^1^ Department of Animal Science Faculty of Agriculture Sanandaj Branch Islamic Azad University Sanandaj Iran; ^2^ Department of Animal Science Faculty of Agricultural and Natural Resources Persian Gulf University Bushehr Iran; ^3^ Department of Biology Faculty of Basic Sciences Kurdistan University Sanandaj Iran; ^4^ Department of Animal Breeding and Genetics University of Veterinary and Animal Sciences Lahore Pakistan; ^5^ Department of Animal Breeding and Genetics University of Agriculture Faisalabad Pakistan

**Keywords:** CAT, GSH‐Px, MDA, SOD, T‐AOC

## Abstract

This study aimed to evaluate the effects of intravenous injection of cysteamine (CS) on body weight (BW), growth hormone (GH), thyroid hormones (TH) secretion, and antioxidant status of growing lambs grazing on mountain pastures. Fifteen lambs (3–4 months of age) were randomly allocated into three experimental groups which received different dosages of CS: 0, 20, and 50 mg/kg BW^−1^. The CS was injected on the 1st, 10th^,^ and 20th days of the experiment to the lambs through the jugular vein. Assessment of plasma concentration of GH and TH hormones was carried out at days 0 (a day before the start of CS injections), 15, and 30 of the experiment. The antioxidant enzymes were measured at the end of the experiment. Lambs were weighed at days 0, 10, 20, and 30 of the experiment. The results showed that treatment and time affected the BW, GH, triiodothyronine (T_3_), and tetraiodothyronine (T_4_) secretion. The intravenous injection of CS increased the BW of growing lambs (*p* < 0.01) and increased the plasma concentration of GH, T3, and T4 (*p* < 0.01). The treatment also enhanced glutathione peroxidase (GSH‐Px; *p* < 0.05) and reduced malondialdehyde concentrations (MDA; *p* < 0.01). Total antioxidant capacity (T‐AOC) level reduced in CS‐1 treatment compared to GC and CS‐2 treatments (*p* < 0.01). The levels of superoxide dismutase (SOD) and catalase (CAT) were not affected by CS. In conclusion, intravenous injection of CS improved BW, GH, and TH concentrations and antioxidant capacity in growing lambs grazing on mountain pastures.

## Abstract 2

In this study, we studied the effect of cysteamine on body weight and secretions of GH and thyroid hormones and the antioxidant capacity of growing lambs in the extensive production system in growing lambs. Generally, we observed the positive effect of cysteamine on growth parameters and antioxidant capacity in growing lambs.

## 1 INTRODUCTION

In Kurdistan province and most of the other regions in Iran, sheep are commonly reared extensively in mountainous and marginal regions. In these systems, feeding management relies on the seasonal availability of pastures as lambs traditionally graze on mountain pastures during spring, summer, and autumn. In such systems, indoor housing takes place only for 3 months in the winter.

The optimum growth performance of lambs is of great economic importance which can be attained by ensuring a proper growth rate throughout the production cycle. The growth hormone (GH) is the most important hormone regulating the growth rate in mammals. Neuroendocrine control of GH secretion is multifactorial, with a balance of stimulatory and inhibitory neurohormones acting on pituitary somatotroph cells (McLeod et al., 1997; Muller et al., 1999; Xiao & Lin, 2003). Somatostatin is recognized as the major inhibitory factor of basal and stimulated GH secretion (McElwain et al., 1999). Promotion of GH secretion and synthesis increases the growth rate (Barnett & Hegarty, 2016) and on the other hand, exogenous brain neuroendocrine factors provide notable growth‐stimulating effects.

Attenuating the action of somatostatin could enhance GH secretion and afterwards increase growth performance (Liu et al., 2009; Sacheck et al., 2004). Likely, active or passive immunization of animals against somatostatin provides an alternative to enhance GH secretion as a means of increasing growth (Dubreuil et al., 1989). In addition, somatostatin has inhibitory effects through various physiological processes in tissues, including neuroendocrine and exocrine secretions from the pancreas and gastrointestinal tract, cell proliferation, nutrient absorption, and splanchnic blood flow (McLeod et al., 1997; C. B. Yang et al., 2005).

The sulfhydryl compound, cysteamine (CS) (mercaptoethylamine), is biologically developed from cysteine metabolism (McLeod et al., 1995). CS is effective in increasing growth, improving feed efficiency, and neutralizing oxidative stress in a range of livestock species (Barnett & Hegarty, 2016). The CS is known to induce not only a decrease in somatostatin and increase in GH release, but also a substantial increase in plasma concentration of T3, T4, insulin, glucose, insulin‐like growth factor‐I (IGF‐I), insulin‐like growth factor‐II (IGH‐II), gastrin, and prolactin in ruminants (Barnett & Hegarty, 2016; Bin et al., 2003; Jia‐dong et al., 2006; Xijie et al., 2000). The CS acts via sulfhydryldisulfide exchange reactions in the glutathione redox cycle, so the sulfhydryl group can theoretically facilitate the synthesis and turnover of glutathione (Liu et al., 2018).

Substantial improvements in performance indices have been observed in response to the administration of CS in mammals, such as improvement in feed efficiency, weight gain, milk yield, and apparent digestibility (Barnett & Hegarty, 2014; Bin et al., 2003; Jia‐dong et al., 2006). The use of CS as a novel growth promoter in animals is affected by different factors such as species of animal, time, dosage and mode of administration, and chemical stability of the feeds.

The GH and thyroid hormones (TH) are the main anabolic hormones that influence the growth rate. To the best of our knowledge, no studies have evaluated the effect of CS administration on the main factors influencing the growth rate in growing lambs reared under an extensive production system. According to CS effects on the growth rate in different species, CS can increase GH through somatostatin depletion and it also has a positive impact on TH secretion and antioxidant status. Therefore, in the present study, the effect of CS administration on growth performance, plasma GH, T3, T4 hormones secretion, and antioxidant capacity was investigated in growing lambs grazed on mountain pasture.

## 2 MATERIALS AND METHODS

### 2.1 Animals and treatments

The present study was conducted from January to August 2019 in the area of Divandareh city (Saral), Kurdistan province, Iran. The Saral region is characterized as semi‐mountainous and mountainous terrain and is located approximately 35 km from Sanandaj city (46°45′ to 46°49′ E and 35°32′N to 35°36′N latitude) at an altitude of 2145 m. During this study, the terrain was mostly used by local sheep and goat breeders for year‐round grazing (Karami et al., 2021). The range of flock size was 200–1500 heads and animals were grazed all day and settled in a defined place at night in the mountain. Our experimental lambs were with the flock and they were not sexually mature. The dominant pasture species in this region were *Bromus tomentellus Boiss*., *Festuca ovina L*., *Ferula Haussknechtii Wolff ex Rech. f*., *Prangos ferulacea (L.) Lindl*., and *Astragalus spp*.

For the present study, 15 Kurdish male lambs were randomly selected within a lamb flock. The age and weight range of lambs were 3–4 months and 25–25.5 kg, respectively. Lambs were distributed in three homogenous treatments (according to age and weight) including control (GC), treatment 2 (CS‐1), and treatment 3 (CS‐2) which received 0 (normal saline), 20, and 50 mg/kg BW^−1^ CS (Sigma Aldrich CN: M6500) through intravenous injection. The concentration of injected CS was selected based on already reported values from the literature (Barnett & Hegarty, 2014; McElwain et al., 1999; McLeod et al., 1995).

The powdered CS was prepared in distilled water immediately before injection to minimize possible oxidation. The special amount of CS for each group was dissolved in the sterile saline solution 0.9% to make the injectable solution. The prepared solution contained 0, 20, and 50 mg/kg of BW^−1^ CS in 0.25 ml of the solution. The duration of the experiment was 30 days. The CS injections were administered three times on the 1st, 10th^,^ and 20th day of the experiment. Animals grazed in the pasture and no additional supplemental diets were given. Animals had ad libitum access to water and feed throughout the whole experimental period.

### 2.2 Growth performance

Initial body weights were measured on day 0 (a day before the beginning of the injections) of the experiment and then the body weights were recorded at 10, 20, and 30 days after the beginning of the experiment.

### 2.3 Plasma hormone and antioxidant assays

Blood samples were collected from each animal via jugular vein on days 0, 15, and 30 to monitor GH and TH secretion. The antioxidant enzymes including glutathione peroxidase (GSH‐Px), superoxide dismutase (SOD), catalase (CAT), malondialdehyde (MDA), and total antioxidant capacity (T‐AOC) were measured at the end of the experiment (day 30). Blood samples were collected into venojects containing ethylene diamine tetra acetic acid and kept on ice. Plasma was separated by centrifugation (1800 × *g*) for 30 min at 4°C and stored at −20°C until the subsequent determination of GH, T3, T4, and antioxidant enzymes. Determination of plasma GH content was performed through a radioimmunoassay (RIA) commercial kit (Daneshyar Noor, Tehran, Iran). Plasma T3 was measured by a competitive enzyme immunoassay (CEI) kit (Padtan Elm Co., Tehran, Iran), and T4 was assessed by CEI kit (Monobind Inc., Lake Forest, CA, USA). All steps of the RIA and CEI methods for GH, T3, and T4 analysis were carried out according to the manufacturer's instructions. The calculated inter‐ and intra‐assay coefficients of variation (CV) were 8.1% and 8.4% for T3 and 12.1% and 12.9% for T4, respectively.

Activities of GSH‐Px, SOD, and CAT were assessed by a commercial kit (Randox Laboratories Ltd., Crumlin, County Antrim, UK) and an autoanalyzer set (Alcyon 300; Abbott lab., Illinois, USA). The measurement of MDA and T‐AOC was also carried out by commercial kits (Randox Laboratories Ltd.).

### 2.4 Statistical analysis

The experimental data of BW, GW, and TH were analyzed by repeated measure design using the general linear model (GLM) of SAS software. The statistical model was as follows:

(1) $$Y_{ijk}=\mu +\alpha_{i}+\beta_{j}+{\left(\alpha \beta \right)}_{ij}+e_{ijk}$$

where *Y_ijk_* is each observation, *μ* is mean, *α_i_* is the effect of treatment *i* (CS), *β_j_* is the effect of time *j*, (*αβ*)*_ij_* is treatment by time interaction effect, and *e_ijk_* is the random error effect.

The antioxidant data were subjected to analysis of variance using the GLM of SAS according to one‐way ANOVA. Differences between treatments were further assessed by Duncan's test and a significant difference was considered if *p* < 0.05.

## 3 RESULTS

### 3.1 Effect of CS on body weight

**FIGURE 1:**
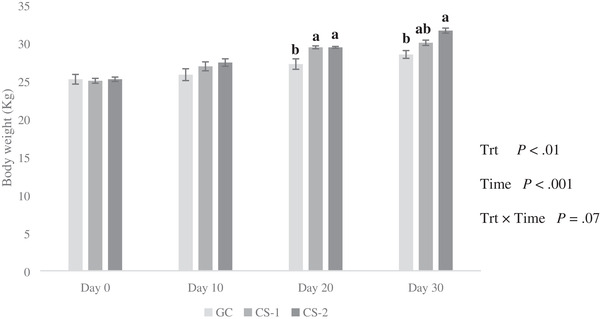
Effect of cysteamine (CS) intravenous injection on the growth performance body weight of growing lambs fed on extensive production system. Bars with different letters are significantly different (*p* < 0.01). Treatments: GC = control treatment (normal saline was administration without CS via i.v. infusion); CS‐1 = 20 mg/kg BW^−1^ CS administrated via i.v. infusion each 10 days; CS‐2 = 50 mg/kg BW^−1^ CS administrated via i.v. infusion each 10 days; Trt = effect of treatment; Time = effect of sampling time; Trt × Time = effect of treatment by sampling time

Repeated measures ANOVA showed that the effects of treatment and time were significant (*p* < 0.01). However, the treatment × time interaction effect was not significant (*p* > 0.05; Figure 1). There were no differences in body weights between groups during the study (*p* > 0.05).

### 3.2 Effect of CS on GH secretion

**FIGURE 2:**
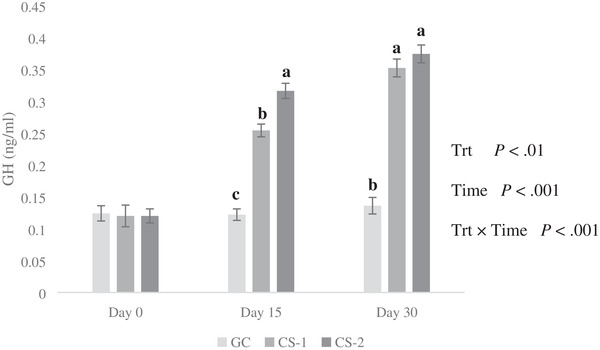
Effect of intravenous injection of cysteamine (CS) on plasma concentrations of growth hormone (GH) in growing lambs fed on extensive production system. Bars with different letters are significantly different (*p* < 0.01). Treatments: GC = control treatment (normal saline was administration without CS via i.v. infusion); CS‐1 = 20 mg/kg BW^−1^ CS administrated via i.v. infusion each 10 days; CS‐2 = 50 mg/kg BW^−1^ CS administrated via i.v. infusion each 10 days; Trt = effect of treatment; Time = effect of sampling time; Trt × Time = effect of treatment by sampling time

The results revealed that the effects of treatment, time, and treatment × time interaction were significant (*p* < 0.01; Figure 2). No significant differences were seen among treatment groups at the beginning of the experiment on day 0 (*p* > 0.05). The CS‐2 group had a higher GH level than CS‐1 on the 15th day of the experiment (*p* < 0.01), but these differences were not sustained at the end of the experiment (Figure 2).

### 3.3 Effect of CS on TH secretion

**FIGURE 3:**
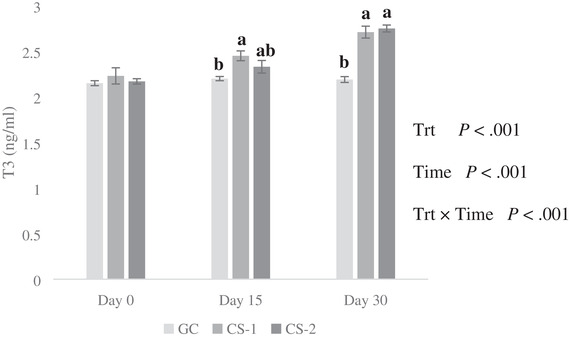
Effect of intravenous injection of cysteamine (CS) on plasma concentrations of T_3_ in growing lambs fed on extensive production system. Bars with different letters are significantly different (*p* < 0.01). Treatments: GC = control treatment (normal saline was administration without CS via i.v. infusion); CS‐1 = 20 mg/kg BW^−1^ CS administrated via i.v. infusion each 10 days; CS‐2 = 50 mg/kg BW^−1^ CS administrated via i.v. infusion each 10 days; Trt = effect of treatment; Time = effect of sampling time; Trt × Time = effect of treatment by sampling time

**FIGURE 4:**
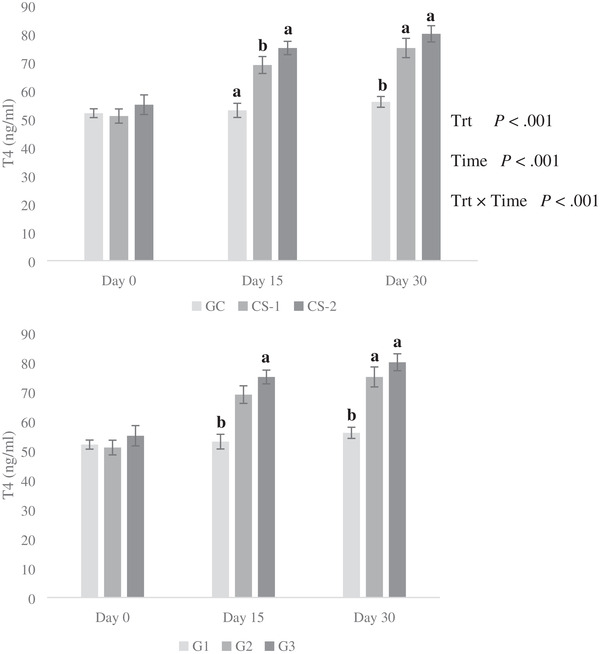
Effect of intravenous injection of cysteamine (CS) on plasma concentrations of T_4_ in growing lambs fed on extensive production system. Treatments: GC = control treatment (normal saline was administration without CS via i.v. infusion); CS‐1 = 20 mg/kg BW^−1^ CS administrated via i.v. infusion each 10 days; CS‐2 = 50 mg/kg BW‐1 CS administrated via i.v. infusion each 10 days; Trt = effect of treatment; Time = effect of sampling time; Trt × Time = effect of treatment by sampling time

The results revealed that effects of treatment, time, and treatment × time interaction were significant on plasma T3 and T4 contents (*p* < 0.01; Figure 3 and Figure 4). There was no difference in T_3_ and T_4_ levels among different groups at the beginning of the experiment (*p* > 0.05; Figures 3 and 4). The CS infusion (both doses) increased the concentration of T_3_ at the end of the experiment (*p* < 0.01; Figure 3), and there was no significant difference between GC and CS‐2 groups on day 15, while the CS‐1 group compared to the control group had significantly higher T3. The CS‐1 and CS‐2 groups had significantly higher concentrations of T_4_ than the control group on 15th and 30th (*p* < 0.01) day of the experiment (Figure 4).

### 3.4 Effect of CS on antioxidant status of growing lambs

Concentrations of antioxidant enzymes in different groups are shown in Table 1. No significant difference was observed in the concentrations of SOD and CAT, but the levels of GSH‐Px (*p* < 0.05) and MDA (*p* < 0.01) were influenced by the administration of CS. The concentration of T‐AOC was significantly decreased in CS‐1 lambs compared to GC lambs; however, it increased in CS‐2. The amount of MDA was reduced in treated lambs, such a decrease was statistically significant in CS‐2, but not in CS‐1 in comparison with the control group (GC).

**TABLE 1:** Effect intravenous injection of cysteamine (CS) on antioxidant enzymes in serum of growing lambs fed on extensive production system

	GC (n = 5)	CS‐1 (n = 5)	CS‐2 (n = 5)	SEM	p‐Value
SOD (U/ml)	14.84	16.42	16.3	0.61	0.5
GSH‐Px (mIU/ml)	53.29 ^b^	62.95^a^	64.29^a^	3.2	<0.05
CAT (IU/ml)	87.92	105.2	114.52	5.83	0.15
T‐AOC (U/ml)	57.06^a^	42.78^b^	60.17^a^	2.3	<0.001
MDA (nmol/ml)	4.52^a^	4.12^ab^	3.31^c^	0.2	<0.001

*Notes*: Means in the same row with no common superscripts differ significantly (*p* < 0.01). Treatments: GC = control treatment (normal saline was administration without CS via i.v. infusion, *n* = 5); CS‐1 = 20 mg/kg BW^−1^ CS administrated via i.v. infusion each 10 days (*n* = 5); CS‐2 = 50 mg/kg BW^−1^ CS administrated via i.v. infusion each 10 days (*n* = 5).

Abbreviations: CAT, catalase; GPX, glutathione peroxidase; MDA, malondialdehyde; SEM, standard error of mean; SOD, superoxide dismutase; T‐AOC, total antioxidant capacity.

## 4 DISCUSSION

In the present study, our findings showed that 20 and 50 mg/kg of BW^−1^ CS had a positive effect on the secretion of GH, TH, and antioxidant status in the growing lams grazed on mountain pasture. Various experiments conducted around the globe have used different forms of CS in various livestock species. Dietary CS supplemented in pigs at 30–700 mg/kg (Bai et al., 2019; Du et al., 2012; C. B. Yang et al., 2005; Zhou et al., 2017), chicken at 60–200 mg/kg (Liu et al., 2018; Nunes et al., 2012; C. M. Yang et al., 2006), and cattle at 5–45 g/day (Wang et al., 2015). In the present study, two doses of CS (20 and 50 mg/kg BW^−1^) were administered to growing lambs raised under an extensive production system. In the previous studies in sheep, CS has been administered through i.v injection at 50 and 100 mg/kg BW^−1^ ((McLeod et al., 1995), and orally at 80 mg/kg BW^−1^(Barnett & Hegarty, 2014)). These studies exhibited that the lower dose (50 mg/kg BW^−1^) of CS had a superior effect; therefore, the lower doses were selected for the present study. Moreover, we investigated the effects of CS on T_3_, T_4_, and antioxidant status on growing lambs grazed on pasture with no supplemental feeds, whereas the condition in the previous studies was not the same.

The infusion of CS (20 and 50 mg/kg BW^−1^ three times) improved the BW of growing lambs under the extensive production system. Although in the present study, we did not measure the feed intake. Some authors suggested that an increase in BW was associated with enhancement of feed intake in pigs, chickens, and sheep (Barnett & Hegarty, 2014; Du et al., 2012; N. Liu et al., 2018). Nevertheless, in these studies, different doses with various supplementation methods (mostly dietary supplementation) increased BW and growth performance indices. In some studies, higher doses of CS had not affected the growth rate, for example, 140 mg/kg in pigs (Lv et al., 2011). The rationale for selecting CS amounts in this study was based upon the fact that lower doses of CS have been shown to increase somatotropin secretion; however, such effect was not observed for higher doses (Barnett & Hegarty, 2014; McElwain et al., 1999; McLeod et al., 1995).

The effects of treatment, time, and their interactions were significant for GH, T3, and T_4_. The interaction effect reveals that the sorting of different treatments was different over time, and CS influenced the concentrations of these hormones divergently. In this study, infusion of 20 and 50 mg/kg BW^−1^ led to an increase in GH concentration. Previous studies in sheep, pigs, chicken, and cattle have shown enhanced levels of GH following CS administration (Dunshea, 2007; McElwain et al., 1999; McLeod et al., 1995; Wang et al., 2015). However, C. B. Yang et al. (2005) reported that CS did not affect GH secretion in pigs. In general, the effect of CS on GH secretion is dose‐dependent and variable. The higher doses of CS have a counter effect on GH secretion (McLeod et al., 1995; C. M. Yang et al., 2006). The increased secretion of GH by systematic depletion of somatostatin has been proposed as the fundamental mechanism underlying the growth‐promoting effect of CS in sheep during the growing and fattening periods (McLeod et al., 1995). The CS increases GH concentration by reducing somatostatin secretion (McLeod et al., 1995), which is usually responsible for suppressing the pituitary secretion of GH (Wang et al., 2015). Moreover, CS increases the activities of protease, amylase, and lipase in the pancreas and small intestinal contents (C. M. Yang et al., 2006). In addition, the growth‐promoting effects of CS may be attributed to the anabolic effects of T3 on the skeletal muscles (C. B. Yang et al., 2005).

In the present study, both doses of CS had a significant effect on the concentration of T3 and T4 in experimental lambs. Similar findings have been reported earlier in broilers (C. M. Yang et al., 2006) and pigs (Jiang et al., 2002; C. B. Yang et al., 2005). C. B. Yang et al. (2005) did not observe any effect of CS supplementation on T4 in finishing pigs. The CS can increase the plasma T3 contents by reducing the inhibitory effect of somatostatin on thyroid‐stimulating hormone and increasing the concentration of GH, which can subsequently increase the concentration of T3 (Kirkwood et al., 1989). The inhibitory effects of somatostatin on pituitary, thyroid, and digestive functions indicate that the suppression of somatostatin by CS could stimulate growth and promote nutrient utilization in growing lambs raised under an extensive production system.

Our results showed that the concentrations of SOD and CAT were not affected by the infusion of CS in growing lambs. CS had a different effect on T‐AOC levels compared to the control group. However, both 20 and 50 mg/kg BW^−1^ decreased the plasma contents MDA, respectively. MDA is usually used as an indicator of oxidative damage in DNA, lipids, and proteins (Nielsen et al., 1997). The GSH‐Px, CAT, and T‐SOD are the most important antioxidant enzymes in the body, which could eliminate unwanted lipid hydroperoxide, •O^2−^, and hydrogen peroxide (Wu et al., 2013). To date, the effects of CS supplementation on antioxidant capacity are not reported in sheep, but reports on other animals are available. Zhou et al. (2017) reported that dietary CS supplementation (142 mg/kg diet) increased the GSH‐Px activity and decreased MDA content, while the concentrations of CAT and T‐SOD were not affected by CS supplementation in finishing pigs. In addition, Lv et al. (2011) suggested that the dietary CS supplementation (70 and 140 mg/kg) increased the T‐AOC while reducing the MDA level in finishing pigs. On the contrary, dietary supplementation of CS had also shown no effect on SOD and MDA in dairy cows (Wang et al., 2015). CS exerts its antioxidant function through its thiol group (Haenen et al., 1989) as the electric charge of the thiol group physically changes the conformation of DNA that decreases the interaction of oxygen radicals with the DNA surface (Vasin, 2014). It enhances the cellular content of glutathione (De‐Matos et al., 1995) that removes free radicals by oxidation of glutathione to glutathione disulphide. At lower concentrations, CS can boost the transport of cysteine into cells that can be used to synthesize glutathione (Barnett & Hegarty, 2016; Mehta et al., 2013).

## 5 CONCLUSIONS

CS administration to lambs grazing on mountain pastures increased their BW. The CS injection also increased the plasma GH and TH contents in addition to exhibiting positive effects on antioxidant enzymes in growing lambs. Hence, the growth and health‐promoting effects of CS are well correlated with its capability to increase GH, TH, and antioxidant capacity in growing lambs reared extensively in the mountainous region.

## AUTHOR CONTRIBUTION

Borhan Shokrollahi: Conceptualization, Data curation, Formal analysis, Funding acquisition, Methodology, Project administration, Resources, Software, Supervision, Writing‐original draft, Writing‐review & editing; Abdolah Fazli: Data curation, Funding acquisition, Investigation, Methodology, Project administration, Resources, Validation, Visualization; Salim Morammazi: Conceptualization, Software, Validation, Writing‐original draft, Writing‐review & editing; Nazila Saadati: CRediT contribution not specified; Hafiz Ishfaq Ahmad: Visualization, Writing‐review & editing; Faizul Hassan: Writing‐review & editing.

## CONFLICT OF INTEREST

The authors declare no conflict of interest.

## ETHICS STATEMNET

The study was undertaken with approval from Sanandaj branch, Islamic Azad University, ethics committee for care and use of animal for research.

### PEER REVIEW

The peer review history for this article is available at https://publons.com/publon/10.1002/vms3.644

## Data Availability

The data that support the findings of this study are available on request from the corresponding author. The data are not publicly available due to privacy or ethical restrictions.
